# Mental health adverse events with cannabis use diagnosed in the Emergency Department: what are we finding now and are our findings accurate?

**DOI:** 10.3389/fpsyt.2023.1093081

**Published:** 2023-05-25

**Authors:** Candice E. Crocker, Jason Emsley, Philip G. Tibbo

**Affiliations:** ^1^Department of Psychiatry, Dalhousie University, Halifax, NS, Canada; ^2^Department of Diagnostic Radiology, Dalhousie University, Halifax, NS, Canada; ^3^Department of Emergency Medicine, Dalhousie University, Halifax, NS, Canada; ^4^IWK Children’s Health Centre, Halifax, NS, Canada; ^5^Department of Psychiatry, Nova Scotia Health, Halifax, NS, Canada

**Keywords:** mental health, adverse event, cannabis (marijuana), Emergency Department use, cannabis legalization

## Abstract

We have previously reviewed the types and numbers of cannabis-associated adverse events that have mental health presentations that are encountered in the Emergency Department. A particular challenge in examining these events is disentangling cannabis use adverse events from adverse events associated with use of multiple recreational substances. Since that review was published, cannabis legalization for recreational use has greatly expanded world-wide and with these changes in the legal climate has come clearer information around the frequency of adverse events seen in the Emergency Department. However, as we examined the current state of the literature, we also examined some of research designs and the biases that may be impacting the validity of the data in this field. The biases both of clinicians and researchers as well as research approaches to studying these events may be impacting our ability to assess the interaction between cannabis and mental health. For example, many of the studies performed examining cannabis-related admissions to the Emergency Department were administrative studies that relied on front line clinicians to identify and attribute that cannabis use was associated with any particular admission. This narrative review provides an overview on what we currently know about mental health adverse events in the Emergency Department with a focus on the mental health impacts both for those with and without a history of mental illness. The evidence that cannabis use can adversely impact genders and sexes differently is also discussed. This review outlines what the most common adverse events related to mental health with cannabis use are; as well as noting the most concerning but much rarer events that have been reported. Additionally, this review suggests a framework for critical evaluation of this field of study going forward.

## 1. Introduction

Cannabis was legalized for recreational use in Canada on 17 October 2018. The reactions to this legislative action appear to be primarily split between two quite divergent viewpoints: positive from the groups who campaigned for cannabis legalization and disappointment from groups involved in treating individuals who experience the negative outcomes of cannabis use. Use of cannabis has quietly increased since legalization in Canada but the enormous business potential expected by the proponents of legalization have also failed to materialize (1). Cannabis use overall in Canada has increased each year since legalization from an estimated 15% of all adults over age 15 in 2017 (pre-legalization) to 25.2% of all adults over 15 in 2021 (2, 3). This increase is similar to what has been seen in other countries that have legalized (as opposed to decriminalized) cannabis use (4). The onset of the COVID-19 pandemic and opioid crisis have stalled what efforts were being made to attempt to inform the general public of the potential harms that cannabis use can pose for some individuals. While it is generally agreed that cannabis adverse events are not common, with increasing tetrahydrocannabinol (THC) concentration coupled with increased frequency of use, this may become a more common issue. For example, the rate of cannabis use disorder, the DSM-5 diagnosis for cannabis dependence or abuse, has increased from an estimated 10% of cannabis users to 22% (5, 6). The need to communicate the risks of cannabis use is ever increasing as there are now 38 states in the United States that have legalized medical cannabis use with 19 legalizing recreational use (4). South Africa, the Seychelles and Ghana have decriminalized cannabis for personal use (7). Other countries such as Canada and Uruguay have fully legalized cannabis use for both recreational and medical use (4). One comprehensive study examined the use of cannabis pre-post legalization in 587 4 year colleges in the United States (US) from 2008 to 2018 comparing cannabis use in college students in states with legalized recreational cannabis to those in states with restricted cannabis approaches and found that past 30 day use increased more in colleges where recreational cannabis was legal (OR = 1.23; 95% CI 1.19–1.28) (8). Here we aggregate the common and uncommon psychiatric adverse events that can be experienced with cannabis use with the hope that this will serve as a resource for Emergency Department (ED) personnel in discussing cannabis use in relation to ED visits for those who have experienced an adverse event related to mental health.

Since publishing our previous paper (9), further studies have examined adverse events related to cannabis use that can be experienced and result in an Emergency Department visit primarily based on administrative data. Papers examining cannabinoid hyperemesis syndrome (CHS) are probably the most common and the most frequently picked up by the media as this is clearly a dramatic adverse event and the related paradoxical effect of increased nausea that can be associated with use of cannabis during pregnancy. An article on the topic in relation to ED impacts was conducted by Andrews and colleagues but like all the cannabis related side effects, this side effect only affects a small proportion of users. However, from pre-post legalization in Canada, the number of ED visits per 100,000 increased from 15 to 21 to 32 in 2020 (10). This still represents a small number of cannabis users. If we frame this as a response to a pharmaceutical that is under consideration for government licensing; this frequency would mean it was considered a rare side effect. If we extend this analysis to examine cannabis adverse events in the same manner as a standard government approved pharmaceutical then the more serious adverse events would be the risk of stroke and some of the lung associated injuries such as hemoptysis which are all more clearly associated with heavy use (11–14). These are what we could consider medical side effects of cannabis use with clear quantitative measures of imaging to show the damage from the event with still more research needing to be done regarding dose relationships and temporal association; but this is outside the scope of this review. What is even more complex to examine and disentangle are the mental health adverse events which we try to address here. Additionally, we examine some of the factors that we believe are potentially complicating analysis of data in this area.

## 2. Methodological approach to this review

This is a narrative review around Emergency Department presentations related to mental health and cannabis use, and it is not a systematic review. We do aim for a balanced approach to show the uncertainties in the literature and indicate areas where we encourage researchers to focus further efforts. The approach we have taken is briefly outlined here. Searches of Pubmed/Medline, Web of Science, and Google scholar were conducted from June 2022 to August 2022 with a focus on papers after October 2020 as this was the end date for our last review on the topic though some prior papers are included to give further context (9). The search terms employed included cannabis or marijuana and Emergency Department and adverse events or mental health or prevalence. Another series of searches was conducted to examine emergency transport, ambulance, and emergency mobile units in conjunction with the term cannabis. We employed the medical subject heading terms for each of the previous terms. This review is focused on effects of cannabis use that result in a need for urgent care and, in particular legal recreational cannabis use on mental health ED presentations. Hence, presentations due to synthetic cannabinoids are not included in this article. Papers located by searching the databases were hand searched for other studies examining mental health impacts associated with confirmed cannabis use in the Emergency Department and emergency transport setting. Published studies from case series to systematic reviews were included in this manuscript. Abstracts were not included.

## 3. Literature update

The research in this field has become more and more defined into two categories. The first is one that examines outcomes is mental health presentations to the ED in individuals who prior to the presentation had no history of a diagnosed mental health disorder. These presentations are often referred to as acute mental health presentations to the ED, although many studies examining acute effects do not record the previous mental health status of the individuals who presented. The other category examines individuals with a previously diagnosed mental health disorder who used cannabis, either acutely or more commonly chronically, and presented to the ED requiring assistance.

### 3.1. Potential impacts of cannabis related ED visits on ED resources

Cannabis related ED visits are not as numerous as visits related to alcohol misuse. However, there is a concern that cannabis related visits may pose a larger resource burden to the health care system. This is a significant concern in today’s healthcare resourcing. One study from Oregon showed only 1.8% of visits to the ED were cannabis-related but for one ED site alone this represented $5.6 million in hospital charges. Cannabis adverse events may represent an all or nothing approach to healthcare needs, if the adverse event reached the level of requiring an ED visit, it was a “significant burden” on hospital resources (15). Another example of how intensive care can be for cannabis intoxicated patients relates to a trauma patient study performed in Los Angeles, California which was not focused on mental health impacts of cannabis, but showed that cannabis use was associated with increased use of mechanical ventilation in trauma patients who had used cannabis (16). This study again represents patient presentations that would pose a significant burden on the healthcare system. A case–control cohort study examining individuals in Ontario, Canada from 2014 to 2017 found that cannabis users had a significantly higher odd of an all-cause ED visit (OR 1.22, 95% CI 1.13 to 1.31) but odds of mortality were not affected (17).

As previously mentioned, since our last examination of this topic there have been considerably more administrative studies conducted on ED visits and cannabis use (9). Earlier administrative reports such as the period of 2012–2016 reported statistically significant increases in the number of ED visits for each year examined; of cannabis related ED visits, with 24.8% were for psychiatric reasons (18). These frequencies seem to be increasing in jurisdictions with cannabis with higher THC content (19, 20). Interestingly, as noted in media interviews product below 24% THC is not of current market interest (1). Increased cannabis use world-wide where cannabis has been legalized may also be contributing to this trend of increasing visits (4). One study actually examined not only the impact of legalization on ED visits related to cannabis use, but also the period of commercialization that occurred about 6 months after legalization in Canada when provincial governments enacted their frameworks for commercial sale of cannabis by a larger retail community. This study by Myran et al. showed that pre-legalization ED visits were increasing but immediate post-legalization the rate leveled off, only to increase again once more commercial outlets were in the marketplace (21). This analysis framework would warn against examining the immediate 6 months pre-and 6 months post-legalization for examining impacts of legalization on ED visits. Another innovative approach to measuring the impact of cannabis legalization on ED service is a study examining the impact of the lottery system for dispensary licenses in Arizona. This study found that Emergency Department visits acutely related to cannabis use rose 45% in the zip codes where a dispensary license was awarded though the visits were not broken out into medical vs. physical health (22).

### 3.2. Studies considering “Cannabis only” ED presentations

Cannabis is often one of several recreational/illicit substances that may be found in an individual patient’s system upon presentation to the Emergency Department. Some studies have attempted to tease apart “cannabis only” clinical presentations. One such study examined cannabis only presentations at an Emergency Department in Switzerland. The study noted that cannabis only presentations overall could be classed as mild but that the group of 186 patients only positive for cannabis had more palpitations (25.3%), anxiety (22.6%), panic attacks (7.5%), and chest pain (14.5%) which was interesting to our group as in our experience the categories of palpitations can overlap with anxiety and panic attacks (23). The classification of psychosis was found in 6.5% of the sample (23). Similarly another retrospective chart review from Michigan covering the time period of November 2018 to October 2020, 39.8% of the individuals presented with an adverse event related to cannabis use that was neuropsychiatric (24). Within this sample of 452 individuals, severe anxiety was the most common presentation at 36.1% followed by altered mental status at 22.3%, suicidal ideation at 14.4%, and hallucinations at 12.8% and psychosis was the presenting complaint in 4.2% of the presentations. This study also showed a longer length of ED stay for neuropsychiatric presentations and not surprisingly, greater odds of a psychiatric admission (24). A similar research design of ICD administrative data but with chart review included, showed visits related to cannabis use increasing year over year in Colorado for psychiatric related chief complaints from 2012 to 2016 with psychiatric codes for both chronic and acute type presentations comprising 63.0% of the visits (18). 75% of the mental health related visits were acute with anxiety being 13.4% of the presentations (*n* = 85) and concerningly, suicide attempt as the next most common at 11.9% (*n* = 75) (18). The discrepancies between these two studies with and without chart review may reflect issues in methodology of one study only using ICD code data where without the “chart check,” the cannabis association is missed.

There is a body of literature not only examining cannabis related ED visits but specifically examining what impact cannabis legalization had on mental health visits to the Emergency Department. The results from these studies vary widely and this may be due to differences in methodological approaches. One administrative database study from an Alberta, Canada ED found a decrease in psychotic diagnoses in the ED over time comparing pre-legalization (2013) to post-legalization (2019). However, there was a significant increase in individuals leaving the ED against medical advice/prior to treatment which could call this result into question (25). An electronic surveillance reporting system used for 19 selected Emergency Departments across Canada showed an annual percent change of 30.1% for all cause cannabis related ED visits for both children and adults between 2015 and 2018 (26). 31.3% were cannabis only presentations (26). A study from a single ED in Ontario, Canada did not show an increase in their cannabis related ED visits comparing the 6 months before and the 6 months after legalization though the age of presentation did vary with individuals between 18 and 29 years showing a 56% increase in cannabis related ED visits over the study periods. The sample size for this study was quite small with 79 cases in the pre-legalization cohort and 94 cases in the post-legalization cohort (27). Pertinent to this discussion, the chief complaint overall for both cohorts was substance abuse (29%), with bizarre behavior next at 16%, hallucinations/delusions were at 6% but unusually, anxiety was the lowest of the mental health codes at 4% of the sample (27). Electronic records from Alberta and Ontario, Canada from 1 April 2015 to 31 December 2019 were used to examine occurrence of psychotic illness associated with cannabis use pre and post-recreational cannabis legalization and found that ED encounters doubled for cannabis-induced psychosis during the time period examined. Using the National Ambulatory Care Reporting System (NACRS), this group found no impact of legalization on occurrence of ED related visits for psychosis in this study with a larger number of encounters examined than some other studies cited here (greater than 200,000 visits) (28). However, this study had a couple of differences from some other administrative studies, only the ICD-10 code for F12.5 cannabis induced psychosis and the ICD-10 related codes for schizophrenia and related disorders were used without inclusion of the hallucinations or delusions codes. This study would likely have a mix of acute psychosis and previously diagnosed with a psychotic disorder and as noted by the authors, studies are lacking to assess the validity of the approach (28). Altogether these studies show that there are measurable numbers of cannabis-associated mental health encounters in the ED but whether legalization was a factor in the increasing rate over time seems unclear.

#### 3.2.1. ED visits related to cannabis use and sex or gender

The increase in cannabis use from 2017 to 2021 in Canada is largely attributable to a significant increase in use by women (2). The UN drug report 2022 also demonstrates that the gender gap in cannabis use is closing world-wide (4). This closing gender gap is also reflected in the results of studies examining the EURO-DEN database of drug involved ED encounters from 36 centers in 24 European countries and in individuals 20 years of age or less, there was no difference in representation of cannabis-related encounters between males and females (29). This cohort has 9.8% of the drug related presentations being cannabis related with only co-ingestion of alcohol allowed for inclusion. An interesting observation from the EURO-DEN cohort, which for all ages is 70% male, was that anxiety was the top clinical feature associated with cannabis intoxication presenting to the ED at 28% of the presentations. However, when broken out by sex, 32.3% of females presented in this manner as compared to 25.4% of males (30). Agitation was classified separately and comprised 23% of the ED presentations with acute psychosis at 9% of the cohort of 4,268 presentations. Patients older than 49 years were less likely to present with anxiety (30). For comparison, the nationwide Emergency Department sample (NEDS) database in the US was examined for cases of cannabis poisoning and for the year 2016, 0.014% of the total ED admissions were cannabis related but these admissions were more likely to meet criteria for various mental illnesses including psychosis, anxiety and mood disorders with females having an association between cannabis toxicity and anxiety (AOR of 2.30) or mood disorder (AOR 2.30) that was significantly higher than the associations seen for males with the same conditions (31). Reasons for difference in the cannabis related presentations between males and females are under study by various approaches with one group examining partnered ED patients showing adverse childhood events being associated with a greater odds of problematic substance use in females (32).

#### 3.2.2. Cannabis presentations and route of administration

There is also evidence that route of exposure may impact what the character of the presentation to the ED will be. The evidence base for this point is not extensive, but it is instructive to consider the issues around the different routes of cannabis administration. One older case series showed hospitalization for cannabis-induced psychosis due to edibles was the outcome in a population of daily cannabis smokers. These individuals reported consuming more than 100 mg of THC prior to the admission and no other substance use reported with only two of the 5 patients having had a previous episode of cannabis induced psychosis (33). This paper highlights that even experienced cannabis users may need further information on the possible dangers of edible cannabis products. In a retrospective chart review done on ED visits in Colorado between 2012 and 2016, among visits attributable to cannabis, encounters associated with inhaled cannabis were more likely to be cannabinoid hyperemesis syndrome (18%) as the top presentation as opposed to oral ingestion which had acute psychiatric symptoms (18%) or intoxication (48%) with edibles accounting for a greater number of ED visits than their sales numbers would suggest (34). A recent retrospective cohort study from seven EDs in Western Michigan, where cannabis was legalized in December 2018, covering the period of November 2018 to July 2020, found 17.1% of ED admissions were related to edibles and that admissions related to edibles increased over the study period post-legalization (35). The consideration of a divergence for medical vs. psychiatric symptomology based on route of ingestion is an area for further study.

The reason for this discrepancy between inhaled and ingested cannabis effects may be two-fold. The first may relate to pharmacokinetics, and a point that many readers will be familiar with, that inhaled cannabis is absorbed with a peak plasma concentration within minutes and has intoxication effects within 15 to 30 min as compared to oral consumption that affects the user’s system within 1–2 h (36). The second point may be a pharmacodynamic one. The inhalation of cannabis bypasses first pass metabolism by the liver whereas oral administration does not. This results in different metabolite levels with different affinities for the cannabinoid receptors as the predominant metabolites in the user’s body. 11-hydroxy-THC, which is also psychoactive, is the predominant metabolite but is seen at higher concentrations after oral ingestion and it has a higher affinity for the CB1 receptor than Delta-9 THC (37, 38). Another point made by Lewis et al., is that cannabis edibles are generally made from cannabis extracts, further increasing the likelihood that the dosing information is not correct on the package, or that the THC content is not homogeneous in the product (35).

#### 3.2.3. ED visits related to cannabis use in individuals with medical authorization or undergoing substance treatment

While the focus is on recreational cannabis, there are also studies examining ED presentations in those with medical cannabis usage. An interesting side note to this topic are two recent surveys of emergency physicians that showed 68.3% of respondents believed that cannabis is medically beneficial (39) and 70.7% agreed that cannabis has medical value (40). ED physicians in the surveys also showed an awareness of the evidence for medical cannabis use for pain and post-chemotherapy vomiting (40). A cohort study from Alberta, Canada examined the short term outcomes for 29,153 individuals with medical authorization to use cannabis and found that within a median time frame of 240 days, 14 patients visited the ED or had cannabis poisoning that resulted in hospitalization and a further 26 individuals visited the ED or were hospitalized for mental health concerns (41). Clearly, this is not a significant rate of adverse events but the study did develop seven predictors of a mental health ED visit for medical cannabis users which included prior poisoning by psychoactive drugs, mental and behavioral disorders due to psychoactive drugs or alcohol, other previous mental health disorders and younger age (41). This suggests factors that could be used to determine who is suitable for a medical cannabis authorization and prior mental health concerns would be a contraindication to medical use.

#### 3.2.4. Cannabis related presentations in those with a diagnosed substance use disorder

The assessment of ED use by individuals with cannabis use can also be examined from the approach of looking at how many ED visits individuals who are in treatment for a cannabis use or related disorder had. One study looked at healthcare utilization overall by individuals in a substance use disorder treatment program and looked at ED utilization by SUD category in Belgium. Individuals with a cannabis use disorder (CUD) had a rate ratio of 2.8 when comparing cases and controls for use of the ED (42). Another study looked at cannabis use disorders which can be associated with chronic cannabis use such as mental illness, addiction, anxiety, or suicidal behaviors as well as chronic physical illnesses such as lung and cardiovascular conditions. This is broader than examining individuals undergoing cessation therapy but as expected multimorbidity associated with cannabis use predicted more ED use (43). However, when compared relatively in another study, ED service use for individuals seeking treatment for a cannabis use disorder was less than that of alcohol use disorder patients and polysubstance users (44).

#### 3.2.5. Cannabis use and suicidality

The role of cannabis-associated adverse events in suicides is still unclear; however, this issue is now more often being addressed in research studies. One example from a retrospective chart review from Michigan from November 2018 to October 2020 found that of the 452 individuals presenting with an adverse event related to cannabis use that was neuropsychiatric in nature, suicidal ideation was seen in 14.4%, and hallucinations at 12.8% (24). In another study, 299 acute psychiatric presentations to the ED from 2012 to 2016 in Colorado were examined, as previously mentioned, and suicide attempts were 75 of the presentations (11.9% of the overall ED presentations related to cannabis) (18). This compiled data, while lacking currently in depth, is concerning not only for the immediate outcome of harm or mortality but also for the work in the field of psychotic disorders that has shown substance-induced psychosis (including cannabis) with self-harm as a feature of the presentation is a predictor for future conversion to psychotic or bipolar disorder (45).

### 3.3. Cannabis use and homicidal or violent presentations to the ED

The most overlooked by the public and quite concerning mental health presentation with cannabis use is individuals who have a severe aggressive adverse reaction to their cannabis intoxication. One study from Switzerland examined cases of violent ED presentations and found 103 cases of violence in 164,846 ED encounters so this can be considered a very rare presentation. However, half of these cases involved cannabis use and overall cannabis was associated with more of the violent cases than was cocaine (46). Also of note, 14 of the cases were associated with domestic violence and 39% of those were cannabis related (46). However, co-use of alcohol was not an exclusion criteria for this study. Another study from Victoria, Australia examined 548 violent events in a regional ED and found that 2% of them were related to cannabis use. The authors of this paper also note that violence was more likely to be associated with cannabis withdrawal than intoxication (47). Homicidal ideation was reported in 3.1% of another study from Michigan (24). Another study from Spain looked at the role of age in cannabis related presentations to the ED and found that agitation, aggression and psychosis were more common in patients over 40 years of age (48). Another study looking at point of care saliva testing for illicit substance use among individuals who required a security response for an unarmed threat in the ED found that 8% of their prospective sample was positive for cannabis and among the entire sample, only 22% reported past 24 h illicit drug use but point of care testing for illicit substances found positive tests were 40.2% of the sample (49). This study illustrates another confound in this body of research of the reliance in many cases of the patient self-reporting their cannabis use.

### 3.4. Mental health issues with intoxication in children

Mental health symptoms, and the potential for permanent changes in brain structure in developing brains with repeated exposures to cannabis, have been well-described in adolescents. However, Emergency Department presentations for cannabis intoxication or poisoning in children tend to include more physical symptoms such as ataxia, lethargy, and tachycardia, and not symptoms related to mental health. We do have evidence that these adverse events are increasing in frequency with one study reporting cannabis-related visits rose from 3.8 per 100,000 in a cohort with an upper age limit of 24 in 2003 to 17.9 per 100,000 in 2017 (50). While the upper age limit of 24 is a classification more of emerging adult than youth or children, it may be instructive to note that the setting for this study was in Canada where medical cannabis, but not recreational cannabis, was legal at the time. However, as has been reported, going through a medical approval phase affects population attitudes toward perception of risk for cannabis use (51). Poisonings in children can be severe though are rarely fatal. The concern is that the long-term impact on the developing brain of having a cannabis poisoning at a young age is not currently known. While it is known that repeated cannabis exposure in youth under 18 years of age is a risk factor for the development of psychosis and may have lasting impact on cognition, it is not clear what the impact of a single large dose of THC might be (52–54). Studies to date have been focused on the immediate outcomes of childhood poisoning with most studies reporting an average age of 3 years for accidental ingestion (21). This is an area for future research.

There are more studies on the impact of cannabis on mental health in the adolescent population since our last review. One recent study using sentinel surveillance of self-harm using the electronic Canadian Hospitals Injury Reporting and Prevention Program from 2011 to 2019 showed an increase of 15.9% per year in self harm with intentional substance-related injuries exceeding unintentional injury cases and 92.3% of the cannabis-related self-harm being in the 10–19 years of age group (55).

### 3.5. Limitations of ICD based studies

Many of these studies were conducted on administrative databases and based on the exclusive use of ICD codes to retrospectively identify cannabis attributable cases. Several groups, ours included, have begun to wonder if this approach is sufficient to accurately identify and track these encounters. ED clinicians may not explicitly use the drug related code, instead opting to use a more symptom related code either as a preference or in the busy atmosphere of an ED use the first code that “fits” the presentation in front of them. One study from Oregon used ICD codes and the electronic medical record with an embedded question asking the clinician to consider if this presentation was cannabis related. This gave 1.6% of classified visits that were cannabis attributable for adults and 0.66% of pediatric visits with cannabis relation but the authors noted that among the charts classified by the question as being cannabis related, only 22% for adults and 17% for pediatric cases had a cannabis related ICD code in the record (15). This suggests there was a disconnect between the entry of a cannabis related ICD code and the association of the presentation with cannabis use. Our work which is in the preliminary analysis stage examining 52, 427 presentations to our three local EDs using ICD-9 codes for the period between October 2018 and June 2020 show 1.7% of presentations being related to cannabis by ICD code but when the charts were hand searched 4.8% were found to be related to cannabis use by the ED encounter chart notes (Crocker, pers. comm). While there are few studies to examine this point, it does raise the question of are we approaching the impacts of cannabis on the ED in the most comprehensive way?

### 3.6. How accurate is our approach to examining ED visits related to cannabis use

How we gauge the impact of recreational and medical cannabis use presenting in the ED may benefit from a bit of re-thinking. High workload demands, a need to address the most immediate health concern and implicit bias may all be playing a role in the quality of the data that is used for much of the research in this field. A recent study in the ED for example examined rates of mistriage and found that roughly 30% of encounters were mistriaged across over 5 million encounters in the United States with groups such as Black Americans more likely to be mistriaged suggesting bias may play a role in the mistriage rates (56). There is a body of literature examining bias in healthcare delivery, with healthcare bias usually being reflected in poorer quality mental healthcare. Individuals with mental illness and addictions experience lower quality of care overall, with these diagnoses identified as a key factor in these negative outcomes (57). There are also studies showing health professionals have an implicit stigma against individuals with mental illness that can lead to poor outcomes for these patients (57–59). However, there is more than one type of stigma and some work has shown that implicit bias predicted over-diagnosis in individuals with mental health training and explicit bias predicted more negative outcomes for patients compared to providers with less mental health experience (60). Bias has been studied in ED personnel primarily with a socioeconomic lens (61). The ED is an environment that is high stress and highly physically demanding at times. While studies that focus on quality of care have examined possible errors to clinical practice with exhaustion in the ED environment, there is also a component of emotional exhaustion which can affect executive function and potentially allow a greater influence of personal bias as a result (62). There may also be, as noted by another group, biases in assigning cannabis use codes to certain racial and ethnic groups which might be related to the frequency of cannabis attributable visits (15). All of this discussion leaves aside the complication that not every patient will report cannabis use in the Emergency possibly due to stigma and given the long half-life of THC in the body, toxicological tests are not always informative. Combined, these factors suggest an examination of the impact of potential bias on ED encounters is required, particularly as it relates to cannabis associated physical and mental health ED presentations.

Further evidence that we may not be accurately tracking use of cannabis in the Emergency Department can be made by inference from the fatality information seen in motor vehicle collisions. The percentage of fatally injured drivers in the United States that had cannabis in their system in 2000 was 9.0%, but by 2018 it had risen to 21.8% in comparison to alcohol involvement which had remained stable (63). Additionally, when trauma patients were assessed, one study found 43% of these patients in 2016 had at least cannabis in their system and that injuries associated with the presence of cannabis were more likely to require mechanical ventilation (16). Another study done in Georgia showed that the odds of dying with cannabis were greater than those of cocaine if presentation to the ED was required (64). This information logically implies that cannabis use is more widely associated with trauma and fatalities and ED presentations than our current ICD code-based studies would suggest.

ED clinicians logically enter the codes for the trauma or symptoms to be urgently dealt with and entering a note on the role of cannabis may not be a priority in that moment. Additionally, the ED is commonly a very busy hospital unit and if triage codes are subject to error then by extension one might consider how accurate ICD code entry may be (56) Further not every clinical case in the ED is simple. For example, the situation with mental health presentations can complicated by an initial uncertainty of whether an anxiety presentation is a reflection of an acute adverse event or a longer standing diagnosis. However, if a drug–drug interaction between cannabis and a psychiatric medication has reduced the effectiveness of that psychiatric medications and this is the underlying cause of the ED presentation, how do we code this case? Many studies being done in the realm of the ED are subject to these complications but these weaknesses and how to address them to improve the accuracy of our findings are rarely discussed. This is why we suggest the new approach that groups such as (18) and our own group are taking of both using ICD codes and searching the electronic medical record are so important (18). Interestingly, Shelton found that ICD codes related to cannabis use were not necessarily attributed to cannabis use whereas our work suggests that the ICD codes are missing the cannabis association and merely representing the symptomatic presentation. These approaches demonstrate the weakness of a strictly administrative code approach for considering the role of substance use such as cannabis in ED visits but also may suggest differences in culture between American and Canadian Emergency Departments. In either case, the wider adoption of electronic charting, and technology to allow searching within these charts, will improve access to visit notes and hopefully add depth to findings in this field.

## 4. Where do we go from here?

Cannabis-associated ED presentations are not numerous, but as the literature base is expanding for this topic, if the cannabis-related presentation reaches the level of requiring urgent care, it is likely to be more complex and costly than alcohol related ones (15). We continue to espouse a view that, like alcohol, cannabis is best used in moderation and it not suitable for everyone. We would encourage public health campaigns to echo this message.

There are several possible approaches being tried to address the problem of increasing cannabis associated ED visits. One approach is brief interventions for cannabis use in the ED which may work for an acute presentation by an occasional user; however, cannabis use disorder has shown to be quite intractable to treatment, and thus these interventions may not be of value for chronic users (65, 66). Another approach is to address planning capacity for mental health and addictions services in the ED setting (67). This would include embedding consultation liaison services in the ED or an adjacent psychiatric emergency service in the ED which is occurring in some locations. Further work is also needed to address the question of whether legalization of cannabis use affects the opioid crisis by reducing opioid-related emergency visits. Overall, the appearance of cannabis related ED visits appears to be continuing to rise and with increased use of edibles and higher THC content, this seems unlikely to change. The total number of these visits may not comprise a high volume of ED presentations but as noted in this review, these presentations may be complex and costly resulting in a greater burden to the healthcare system than the number of encounters might suggest.

There are also concerns noted here that we may be underestimating the extent of the problem due to a variety of possible reasons related to entering ICD codes not reflecting the involvement of cannabis use in the encounter. Future research should address this potential problem through methods such as ICD study validation by cross-checking the medical record for ED encounter notes to ensure cannabis related encounters are not being overlooked. While we work directly in a team of both emergency room clinicians and psychiatry clinicians and researcher, this is not always the case. It would benefit this research field if more researchers worked directly with ED staff to educate or explain the importance of the use of these codes to accurately reflect drug involvement if we are to base our healthcare planning on these approaches.

We hope this review provides information to ED clinicians on the likely impacts of cannabis on their practice and serves as a reference when addressing a patient’s contentions that their cannabis use is harmless. There is an argument to be made that this is the case for the majority of occasional cannabis users but like any drug, cannabis will have adverse effects on some individuals who take it.

## Author contributions

CC wrote the first draft and compiled the edits. PT and JE edited drafts. All authors contributed to the article and approved the submitted version.

## Conflict of interest

The authors declare that the research was conducted in the absence of any commercial or financial relationships that could be construed as a potential conflict of interest.

## Publisher’s note

All claims expressed in this article are solely those of the authors and do not necessarily represent those of their affiliated organizations, or those of the publisher, the editors and the reviewers. Any product that may be evaluated in this article, or claim that may be made by its manufacturer, is not guaranteed or endorsed by the publisher.

## References

[1] Solman P. Marijuana has become big business. So why are small growers struggling to survive? PBS News Hour. (2019). Available at: https://www.pbs.org/newshour/show/marijuana-has-become-big-business-so-why-are-small-growers-struggling-to-survive

[2] Canada G. o.. Canadian Cannabis survey results blog, 2021. (2021). Available at: https://health-infobase.canada.ca/cannabis/

[3] Drugs C. T. A. A. Canadian tobacco alcohol and Drugs (CTADS): 2017 summary. Health Canada. (2017). Available at: https://www.canada.ca/en/health-canada/services/canadian-alcohol-drugs-survey/2017-summary/2017-detailed-tables.html

[4] UNODC. World Drug Report 2022. (2022). Available at: https://www.unodc.org/unodc/en/data-and-analysis/world-drug-report-2022.html

[5] Hall W Degenhardt L. The adverse health effects of chronic cannabis use. Drug Test Anal. (2014) 6:39–45. doi: 10.1002/dta.1506 23836598

[6] Leung J Chan GCK Hides L Hall WD. What is the prevalence and risk of cannabis use disorders among people who use cannabis? A systematic review and meta-analysis. Addict Behav. (2020) 109:106479. doi: 10.1016/j.addbeh.2020.106479, PMID: 32485547

[7] Kitchen C Kabba JA Fang Y. Status and impacts of recreational and medicinal Cannabis policies in Africa: a systematic review and thematic analysis of published and "gray" literature. Cannabis Cannabinoid Res. (2022) 7:239–61. doi: 10.1089/can.2021.0110, PMID: 34986005 PMC9225416

[8] Bae H Kerr DCR. Marijuana use trends among college students in states with and without legalization of recreational use: initial and longer-term changes from 2008 to 2018. Addiction. (2020) 115:1115–24. doi: 10.1111/add.14939, PMID: 31833119

[9] Crocker CE Carter AJE Emsley JG Magee K Atkinson P Tibbo PG. When Cannabis use Goes wrong: mental health side effects of Cannabis use that present to emergency services. Front Psych. (2021) 12:640222. doi: 10.3389/fpsyt.2021.640222, PMID: 33658953 PMC7917124

[10] Andrews CN Rehak R Woo M Walker I Ma C Forbes N. Cannabinoid hyperemesis syndrome in North America: evaluation of health burden and treatment prevalence. Aliment Pharmacol Ther. (2022) 56:1532–42. doi: 10.1111/apt.17265, PMID: 36307209

[11] Dutta T Ryan KA Thompson O Lopez H Fecteau N Sparks MJ. Marijuana use and the risk of early ischemic stroke. Stroke. (2021) 52:3184–90. doi: 10.1161/STROKEAHA.120.032811 34266309 PMC8478805

[12] Page RL Allen LA Kloner RA Carriker CR Martel C Morris AA. Medical marijuana, recreational Cannabis, and cardiovascular health: a scientific statement from the American Heart Association. Circulation. (2020) 142:e131–52. doi: 10.1161/CIR.0000000000000883, PMID: 32752884

[13] Swetlik C Migdady I Hasan LZ Buletko AB Price C Cho SM. Cannabis use and stroke: does a risk exist? J Addict Med. (2022) 16:208–15. doi: 10.1097/adm.0000000000000870, PMID: 34001774

[14] Toquet S Cousson J Choiselle N Gozalo C Giusti D Bani-Sadr F. Alveolar hemorrhage due to marijuana smoking using water pipe made with plastic bottle: case report and narrative review of the literature. Inhal Toxicol. (2021) 33:168–76. doi: 10.1080/08958378.2021.1939465, PMID: 34180335

[15] Hendrickson RG Dilley JA Hedberg K Jeanne TL Love JS Thompson JA. The burden of cannabis-attributed pediatric and adult Emergency Department visits. Acad Emerg Med. (2021) 28:1444–7. doi: 10.1111/acem.14275, PMID: 33966297 PMC11215941

[16] Banks K Biswas S Wong M Byerly S Clark D Lam L. Cannabis use is associated with increased mechanical ventilation and polysubstance use in trauma patients. Am Surg. (2019) 85:226–9. doi: 10.1177/000313481908500234, PMID: 30819304

[17] Vozoris NT Zhu J Ryan CM Chow C-W To, T. Cannabis use and risks of respiratory and all-cause morbidity and mortality: a population-based, data-linkage, cohort study. BMJ Open Respir Res. (2022) 9:e001216. doi: 10.1136/bmjresp-2022-001216, PMID: 35760496 PMC9240874

[18] Shelton SK Mills E Saben JL Devivo M Williamson K Abbott D. Why do patients come to the Emergency Department after using cannabis? Clin Toxicol. (2019) 58:453–9. doi: 10.1080/15563650.2019.1657582, PMID: 31526057 PMC7073292

[19] Roehler DR Hoots BE Holland KM Baldwin GT Vivolo-Kantor AM. Trends and characteristics of cannabis-associated Emergency Department visits in the United States, 2006–2018. Drug Alcohol Depend. (2022) 232:109288. doi: 10.1016/j.drugalcdep.2022.109288, PMID: 35033959 PMC9885359

[20] Shen JJ Shan G Kim PC Yoo JW Dodge-Francis C Lee Y-J. Trends and related factors of Cannabis-associated Emergency Department visits in the United States. J Addict Med. (2019) 13:193–200. doi: 10.1097/ADM.0000000000000479, PMID: 30418337

[21] Myran DT Cantor N Finkelstein Y Pugliese M Guttmann A Jesseman R. Unintentional pediatric Cannabis exposures after legalization of recreational Cannabis in Canada. JAMA Netw Open. (2022) 5:e2142521–1. doi: 10.1001/jamanetworkopen.2021.42521, PMID: 34994796 PMC8742190

[22] Conyers G Ayres I. A lottery test of the effect of dispensaries on emergency room visits in Arizona. Health Econ. (2020) 29:854–64. doi: 10.1002/hec.4013, PMID: 32548868

[23] Schmid Y Scholz I Mueller L Exadaktylos AK Ceschi A Liechti ME. Emergency Department presentations related to acute toxicity following recreational use of cannabis products in Switzerland. Drug Alcohol Depend. (2020) 206:107726. doi: 10.1016/j.drugalcdep.2019.107726, PMID: 31735534

[24] Leach E Fomum Mugri LB Keung MY Ouellette L Fleeger T Sapp T. Neuropsychiatric effects of cannabis toxicity in the Emergency Department: a community-based study. Am J Emerg Med. (2022) 56:375–7. doi: 10.1016/j.ajem.2021.10.053, PMID: 34763961

[25] Yeung MEM Weaver CG Janz K Haines-Saah R Lang E. Clearing the air: a study of cannabis-related presentations to urban Alberta emergency departments following legalization. CJEM. (2020) 22:776–83. doi: 10.1017/cem.2020.384 32616094

[26] Champagne AS McFaull SR Thompson W Bang F. Surveillance from the high ground: sentinel surveillance of injuries and poisonings associated with cannabis. Health Promot Chronic Dis Prev Can. (2020) 40:184–92. doi: 10.24095/hpcdp.40.5/6.07, PMID: 32529978 PMC7367429

[27] Baraniecki R Panchal P Malhotra DD Aliferis A Zia Z. Acute cannabis intoxication in the Emergency Department: the effect of legalization. BMC Emerg Med. (2021) 21:32. doi: 10.1186/s12873-021-00428-0, PMID: 33731003 PMC7968301

[28] Callaghan RC Sanches M Murray RM Konefal S Maloney-Hall B Kish SJ. Associations between Canada's Cannabis legalization and Emergency Department presentations for transient Cannabis-induced psychosis and schizophrenia conditions: Ontario and Alberta, 2015-2019. Can J Psychiatr. (2022) 67:616–25. doi: 10.1177/07067437211070650, PMID: 35019734 PMC9301152

[29] Miró Ò Waring WS Dargan PI Wood DM Dines AM Yates C. Variation of drugs involved in acute drug toxicity presentations based on age and sex: an epidemiological approach based on European emergency departments. Clin Toxicol. (2021) 59:896–904. doi: 10.1080/15563650.2021.1884693, PMID: 33724118

[30] Schmid Y Galicia M Vogt SB Liechti ME Burillo-Putze G Dargan PI. Differences in clinical features associated with cannabis intoxication in presentations to European emergency departments according to patient age and sex. Clin Toxicol. (2022) 60:912–9. doi: 10.1080/15563650.2022.2060116, PMID: 35404194

[31] Salas-Wright CP Carbone JT Holzer KJ Vaughn MG. Prevalence and correlates of cannabis poisoning diagnosis in a National Emergency Department sample. Drug Alcohol Depend. (2019) 204:107564. doi: 10.1016/j.drugalcdep.2019.107564, PMID: 31568933 PMC6887107

[32] Cunradi CB Caetano R Alter HJ Ponicki WR. Adverse childhood experiences are associated with at-risk drinking, cannabis and illicit drug use in females but not males: an Emergency Department study. Am J Drug Alcohol Abuse. (2020) 46:739–48. doi: 10.1080/00952990.2020.1823989, PMID: 33186088 PMC8432740

[33] Hudak M Severn D Nordstrom K. Edible Cannabis-induced psychosis: intoxication and beyond. Am J Psychiatry. (2015) 172:911–2. doi: 10.1176/appi.ajp.2015.15030358, PMID: 26324307

[34] Monte AA Shelton SK Mills E Saben J Hopkinson A Sonn B. Acute illness associated with Cannabis use, by route of exposure: an observational study acute illness associated with Cannabis use, by route of exposure. Ann Intern Med. (2019) 170:531–7. doi: 10.7326/m18-2809, PMID: 30909297 PMC6788289

[35] Lewis B Fleeger T Judge B Riley B Jones JS. Acute toxicity associated with cannabis edibles following decriminalization of marijuana in Michigan. Am J Emerg Med. (2021) 46:732–5. doi: 10.1016/j.ajem.2020.09.077, PMID: 33036859

[36] Wong KU Baum CR. Acute Cannabis toxicity. Pediatr Emerg Care. (2019) 35:799–804. doi: 10.1097/PEC.0000000000001970 31688799

[37] Hollister LE Gillespie HK Ohlsson A Lindgren JE Wahlen A Agurell S. Do plasma concentrations of delta 9-tetrahydrocannabinol reflect the degree of intoxication? J Clin Pharmacol. (1981) 21:171s–7s. doi: 10.1002/j.1552-4604.1981.tb02593.x, PMID: 6271822

[38] Sharma P Murthy P Bharath MS. Chemistry, metabolism, and toxicology of cannabis: clinical implications. Iran J Psychiatry. (2012) 7:149–56. PMID: 23408483 PMC3570572

[39] Takakuwa KM Shofer FS Schears RM. The practical knowledge, experience and beliefs of US emergency medicine physicians regarding medical Cannabis: a national survey. Am J Emerg Med. (2020) 38:1952–4. doi: 10.1016/j.ajem.2020.01.059, PMID: 32067838

[40] Takakuwa KM Schears RM. Indications and preference considerations for using medical Cannabis in an Emergency Department: a National Survey. Am J Emerg Med. (2021) 45:513–5. doi: 10.1016/j.ajem.2020.07.005, PMID: 32682602

[41] Zongo A Lee C Dyck JRB El-Mourad J Hyshka E Hanlon JG. Incidence and predictors of Cannabis-related poisoning and mental and behavioral disorders among patients with medical Cannabis authorization: a cohort study. Subst Use Misuse. (2022) 57:1633–41. doi: 10.1080/10826084.2022.2102193, PMID: 35866679

[42] Van Baelen L Plettinckx E Antoine J De Ridder K Devleesschauwer B Gremeaux L. Use of health care services by people with substance use disorders in Belgium: a register-based cohort study. Arch Public Health. (2021) 79:112. doi: 10.1186/s13690-021-00620-5, PMID: 34162427 PMC8220672

[43] Fleury MJ Grenier G Cao Z Huynh C. Predictors of no, low and frequent Emergency Department use for any medical reason among patients with cannabis-related disorders attending Quebec (Canada) addiction treatment centres. Drug Alcohol Rev. (2022) 41:1136–51. doi: 10.1111/dar.13451, PMID: 35266240

[44] Armoon B Grenier G Cao Z Huynh C Fleury MJ. Frequencies of Emergency Department use and hospitalization comparing patients with different types of substance or polysubstance-related disorders. Subst Abuse Treat Prev Policy. (2021) 16:89. doi: 10.1186/s13011-021-00421-7, PMID: 34922562 PMC8684146

[45] Starzer MSK Nordentoft M Hjorthoj C. Rates and predictors of conversion to schizophrenia or bipolar disorder following substance-induced psychosis. Am J Psychiatry. (2018) 175:343–50. doi: 10.1176/appi.ajp.2017.17020223, PMID: 29179576

[46] Liakoni E Gartwyl F Ricklin M Exadaktylos AK. Psychoactive substances and violent offences: a retrospective analysis of presentations to an urban Emergency Department in Switzerland. PLoS One. (2018) 13:e0195234. doi: 10.1371/journal.pone.0195234 29596473 PMC5875877

[47] Kleissl-Muir S Raymond A Rahman MA. Analysis of patient related violence in a regional Emergency Department in Victoria. Australia Australas Emerg Care. (2019) 22:126–31. doi: 10.1016/j.auec.2019.01.006, PMID: 31042524

[48] Burillo-Putze G Ibrahim-Ach D Galicia M Supervia A Martinez-Sanchez L Ortega Perez J. Clinical manifestations and serious adverse effects after cannabis use: role of age according to sex and coingestion of alcohol. Emergencias. (2022) 34:275–81. 35833766

[49] Gerdtz M Yap CY Daniel C Knott JC Kelly P Braitberg G. Prevalence of illicit substance use among patients presenting to the Emergency Department with acute behavioural disturbance: rapid point-of-care saliva screening. Emerg Med Australas. (2020) 32:473–80. doi: 10.1111/1742-6723.13441, PMID: 31927789

[50] Bechard M Cloutier P Lima I Salamatmanesh M Zemek R Bhatt M. Cannabis-related Emergency Department visits by youths and their outcomes in Ontario: a trend analysis. CMAJ Open. (2022) 10:E100–8. doi: 10.9778/cmajo.20210142, PMID: 35135825 PMC9259464

[51] Pacula R Jacobson M Maksabedian EJ. In the weeds: a baseline view of Cannabis use among legalizing states and their Neighbours. Addiction. (2016) 111:973–80. doi: 10.1111/add.13282, PMID: 26687431 PMC5216038

[52] Di Forti M Sallis H Allegri F Trotta A Ferraro L Stilo SA. Daily use, especially of high-potency Cannabis, drives the earlier onset of psychosis in Cannabis users. Schizophr Bull. (2014) 40:1509–17. doi: 10.1093/schbul/sbt181, PMID: 24345517 PMC4193693

[53] Hjorthøj C Larsen MO Starzer MSK Nordentoft M. Annual incidence of cannabis-induced psychosis, other substance-induced psychoses and dually diagnosed schizophrenia and cannabis use disorder in Denmark from 1994 to 2016. Psychol Med. (2021) 51:617–22. doi: 10.1017/S0033291719003532, PMID: 31839011

[54] Kroon E Kuhns L Cousijn J. The short-term and long-term effects of cannabis on cognition: recent advances in the field. Curr Opin Psychol. (2021) 38:49–55. doi: 10.1016/j.copsyc.2020.07.005, PMID: 32823178

[55] Campeau A Champagne AS McFaull SR. Sentinel surveillance of substance-related self-harm in Canadian emergency departments, 2011-19. BMC Public Health. (2022) 22:974. doi: 10.1186/s12889-022-13287-6, PMID: 35568831 PMC9107222

[56] Sax DR Warton EM Mark DG Vinson DR Kene MV Ballard DW. Evaluation of the emergency severity index in US Emergency Departments for the rate of Mistriage. JAMA Netw Open. (2023) 6:e233404–4. doi: 10.1001/jamanetworkopen.2023.3404, PMID: 36930151 PMC10024207

[57] Knaak S Mantler E Szeto A. Mental illness-related stigma in healthcare: barriers to access and care and evidence-based solutions. Healthc Manage Forum. (2017) 30:111–6. doi: 10.1177/0840470416679413, PMID: 28929889 PMC5347358

[58] Hayes RD Chang CK Fernandes A Broadbent M Lee W Hotopf M. Associations between substance use disorder sub-groups, life expectancy and all-cause mortality in a large British specialist mental healthcare service. Drug Alcohol Depend. (2011) 118:56–61. doi: 10.1016/j.drugalcdep.2011.02.021, PMID: 21440382

[59] Ross LE Vigod S Wishart J Waese M Spence JD Oliver J. Barriers and facilitators to primary care for people with mental health and/or substance use issues: a qualitative study. BMC Fam Pract. (2015) 16:135. doi: 10.1186/s12875-015-0353-3, PMID: 26463083 PMC4604001

[60] Peris TS Teachman BA Nosek BA. Implicit and explicit stigma of mental illness: links to clinical care. J Nerv Ment Dis. (2008) 196:752–60. doi: 10.1097/NMD.0b013e3181879dfd 18852619

[61] Turner AJ Francetic I Watkinson R Gillibrand S Sutton M. Socioeconomic inequality in access to timely and appropriate care in Emergency Departments. J Health Econ. (2022) 85:102668. doi: 10.1016/j.jhealeco.2022.102668, PMID: 35964420

[62] Feuerhahn N Stamov-Roßnagel C Wolfram M Bellingrath S Kudielka BM. Emotional exhaustion and cognitive performance in apparently healthy teachers: a longitudinal multi-source study. Stress Health. (2013) 29:297–306. doi: 10.1002/smi.2467, PMID: 23086898

[63] Lira MC Heeren TC Buczek M Blanchette JG Smart R Pacula RL. Trends in Cannabis involvement and risk of alcohol involvement in motor vehicle crash fatalities in the United States, 2000–2018. Am J Public Health. (2021) 111:1976–85. doi: 10.2105/ajph.2021.306466, PMID: 34709858 PMC8630490

[64] Gilmore D Zorland J Akin J Johnson JA Emshoff JG Kuperminc GP. Mortality risk in a sample of Emergency Department patients who use cocaine with alcohol and/or cannabis. Subst Abus. (2017) 39:266–70. doi: 10.1080/08897077.2017.1389799 28991520

[65] Halladay J Scherer J MacKillop J Woock R Petker T Linton V. Brief interventions for cannabis use in emerging adults: a systematic review, meta-analysis, and evidence map. Drug Alcohol Depend. (2019) 204:107565. doi: 10.1016/j.drugalcdep.2019.107565, PMID: 31751868

[66] Imtiaz S Roerecke M Kurdyak P Samokhvalov AV Hasan OSM Rehm J. Brief interventions for Cannabis use in healthcare settings: systematic review and Meta-analyses of randomized trials. J Addict Med. (2020) 14:78–88. doi: 10.1097/adm.0000000000000527, PMID: 32012140

[67] Baia Medeiros DT Hahn-Goldberg S Aleman DM O’Connor E. Planning capacity for mental health and addiction Services in the Emergency Department: a discrete-event simulation approach. J Healthc Engineer. (2019) 2019:8973515–1. doi: 10.1155/2019/8973515, PMID: 31281618 PMC6589296

